# Insulin Granule-Loaded MicroPlates for Modulating Blood Glucose Levels in Type-1 Diabetes

**DOI:** 10.1021/acsami.1c16768

**Published:** 2021-11-09

**Authors:** Rosita Primavera, Elena Bellotti, Daniele Di Mascolo, Martina Di Francesco, Jing Wang, Bhavesh D. Kevadiya, Angelo De Pascale, Avnesh S. Thakor, Paolo Decuzzi

**Affiliations:** † Laboratory of Nanotechnology for Precision Medicine, Fondazione Istituto Italiano di Tecnologia, Via Morego 30, Genoa 16163, Italy; ‡ Interventional Regenerative Medicine and Imaging Laboratory, Department of Radiology, Stanford University, Palo Alto, California 94304, United States; § Unit of Endocrinology, Department of Internal Medicine & Medical Specialist (DIMI), University of Genoa, 16136 Genoa, Italy

**Keywords:** microfabrication, drug delivery, insulin granules, diabetes, microparticles

## Abstract

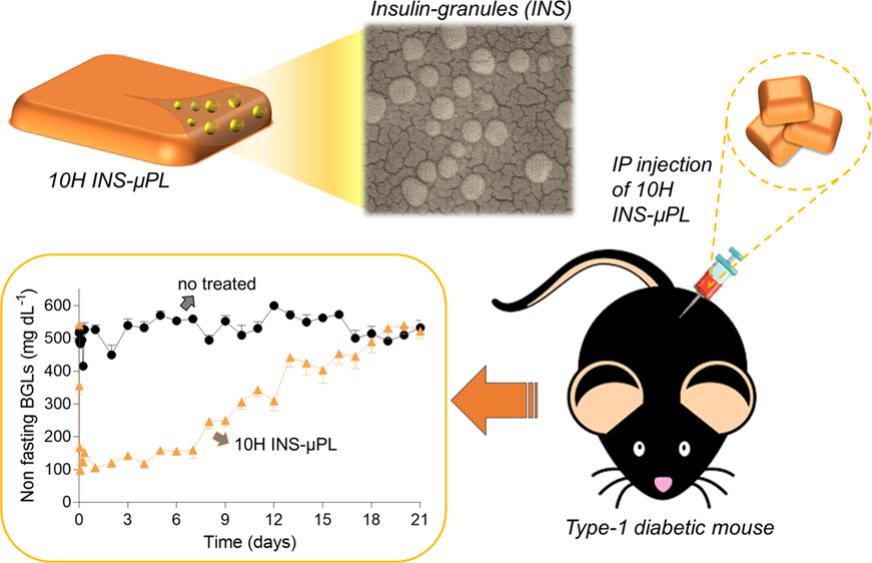

Type-1 diabetes (T1DM) is a chronic metabolic disorder resulting from the autoimmune destruction of β cells. The current standard of care requires multiple, daily injections of insulin and accurate monitoring of blood glucose levels (BGLs); in some cases, this results in diminished patient compliance and increased risk of hypoglycemia. Herein, we engineered hierarchically structured particles comprising a poly(lactic-*co*-glycolic) acid (PLGA) prismatic matrix, with a 20 × 20 μm base, encapsulating 200 nm insulin granules. Five configurations of these insulin-microPlates (INS-μPLs) were realized with different heights (5, 10, and 20 μm) and PLGA contents (10, 40, and, 60 mg). After detailed physicochemical and biopharmacological characterizations, the tissue-compliant 10H INS-μPL, realized with 10 mg of PLGA, presented the most effective release profile with ∼50% of the loaded insulin delivered at 4 weeks. In diabetic mice, a single 10H INS-μPL intraperitoneal deposition reduced BGLs to that of healthy mice within 1 h post-implantation (167.4 ± 49.0 vs 140.0 ± 9.2 mg/dL, respectively) and supported normoglycemic conditions for about 2 weeks. Furthermore, following the glucose challenge, diabetic mice implanted with 10H INS-μPL successfully regained glycemic control with a significant reduction in AUC_0–120min_ (799.9 ± 134.83 vs 2234.60 ± 82.72 mg/dL) and increased insulin levels at 7 days post-implantation (1.14 ± 0.11 vs 0.38 ± 0.02 ng/mL), as compared to untreated diabetic mice. Collectively, these results demonstrate that INS-μPLs are a promising platform for the treatment of T1DM to be further optimized with the integration of smart glucose sensors.

## Introduction

Type-1 diabetes (T1DM) is a chronic metabolic disorder characterized by elevated blood glucose levels (BGLs).^1^ T1DM affects 30 million people globally and results from a cell-mediated autoimmune destruction of the insulin-producing pancreatic β cells.^2^ Insulin administration is considered the main approach for treating T1DM and is also often used in advanced type-2 diabetes mellitus (T2DM).^3^ Before the 1980s, insulin for clinical applications was obtained from the porcine or bovine pancreas, while now it can be created chemically using recombinant DNA technology and appears identical to human insulin.^4,5^ Currently, human insulin formulations available on the market are classified based on their time of action and include rapid-acting insulin (insulin lispro, aspart, and glulisine), intermediate-acting insulin (NPH-insulin and insulin lente), and long-acting insulin (insulin ultralente, glargine, and detemir).^6^ These different insulin formulations are usually administered via subcutaneous injections at different sites (i.e., upper arms, tights, buttocks, and abdomen).^7^ The multiple daily injections of insulin, which are often required to modulate BGLs, are a continuous threat to patient compliance and increase the risk of hypoglycemia possibly causing cardiac arrhytmia, acute coronary syndrome, coma, brain damage, or even death. Furthermore, the improper dosages of insulin can also result in excessive fluctuations of BGLs that, if chronic, result in severe complications (i.e., retinopathy, nephropathy, cardiovascular disease, and diabetic foot), in addition to many other comorbidities.^8−10^

To improve the life quality of diabetics, different approaches have been studied to control and facilitate insulin delivery. For instance, polymeric drug delivery systems (i.e., microspheres,^11,12^ nanoparticles,^13,14^ and hydrogels^14^) and lipid-based systems (i.e., liposomes^15,16^ and solid lipid nanoparticles^17−19^) have been suggested and investigated in preclinical and clinical trials to achieve non-invasive administration including oral or nasal or pulmonary or transdermal and a controlled release of insulin.^20,21^ The suggested platforms have been found to be beneficial in many aspects, such as protecting drugs from enzymatic degradation, improving their stability, enhancing the half-life in the body circulation, overcoming different physical, chemical, and biological barriers in vivo, and increasing bioavailability and therapeutic efficacy. However, during the preparation of these platforms, insulin can be easily damaged. For example, the use of chemicals (i.e., organic solvents), dehydration (i.e., freeze drying), high shear forces (i.e., vortex mixing), organic–aqueous interfaces, and hydrophobic contacts between insulin and the polymer can cause alterations in the structure and a loss of its bioactivity.^22−25^ Moreover, often these techniques are difficult to scale-up for industrial production. Despite these findings, the inability to obtain an optimal formulation that can match the needs of individual diabetic patients has led to innovative self-regulated insulin delivery systems that mimic native insulin production from a healthy pancreas.^20,26,27^ These pancreas-like systems are able to release insulin in response to glucose changes and include insulin pumps or systems that have glucose-sensing elements and are able to trigger insulin release (i.e., glucose oxidase, boronic acid derivatives, and concavalin A).^20,28−31^ However, many challenges remain unaddressed including slow response rate, lack of glucose specificity, instability, acute and long-term toxicity, and short-term efficacy.^26,30^

In this work, hierarchically structured polymeric particles carrying insulin granules, called insulin-microPlates (INS-μPLs), are first introduced with their unique geometrical attributes. INS-μPLs are obtained via a multi-step, top-down fabrication process and result in prismatic microparticles made out of the FDA-approved, biodegradable polymer poly(lactic-*co*-glycolic) acid (PLGA). Spherical, 200 nm insulin granules are uniformly dispersed within the polymeric matrix that protects them from exposure to biological fluids and consequent rapid dissolution. We propose five different configurations of microparticles made with different thicknesses and PLGA concentrations, namely, 5 μm thick with 10 mg of PLGA (5H INS-μPL); 10 μm thick with 10 mg of PLGA (10H INS-μPL); and 20 μm thick with 10, 40, and 60 mg of PLGA (20H INS-μPL with either 10, 40, or 60 mg of PLGA). These five different microparticles are characterized for their physicochemical and biopharmacological properties to identify the configuration with the most suitable properties that is, eventually tested in murine models of T1DM [streptozotocin (STZ)-treated C57BL/6].

## Results and Discussion

### Fabrication, Assembly, and Physico-Chemical Characterization of INS-μPLs

**Figure 1:**
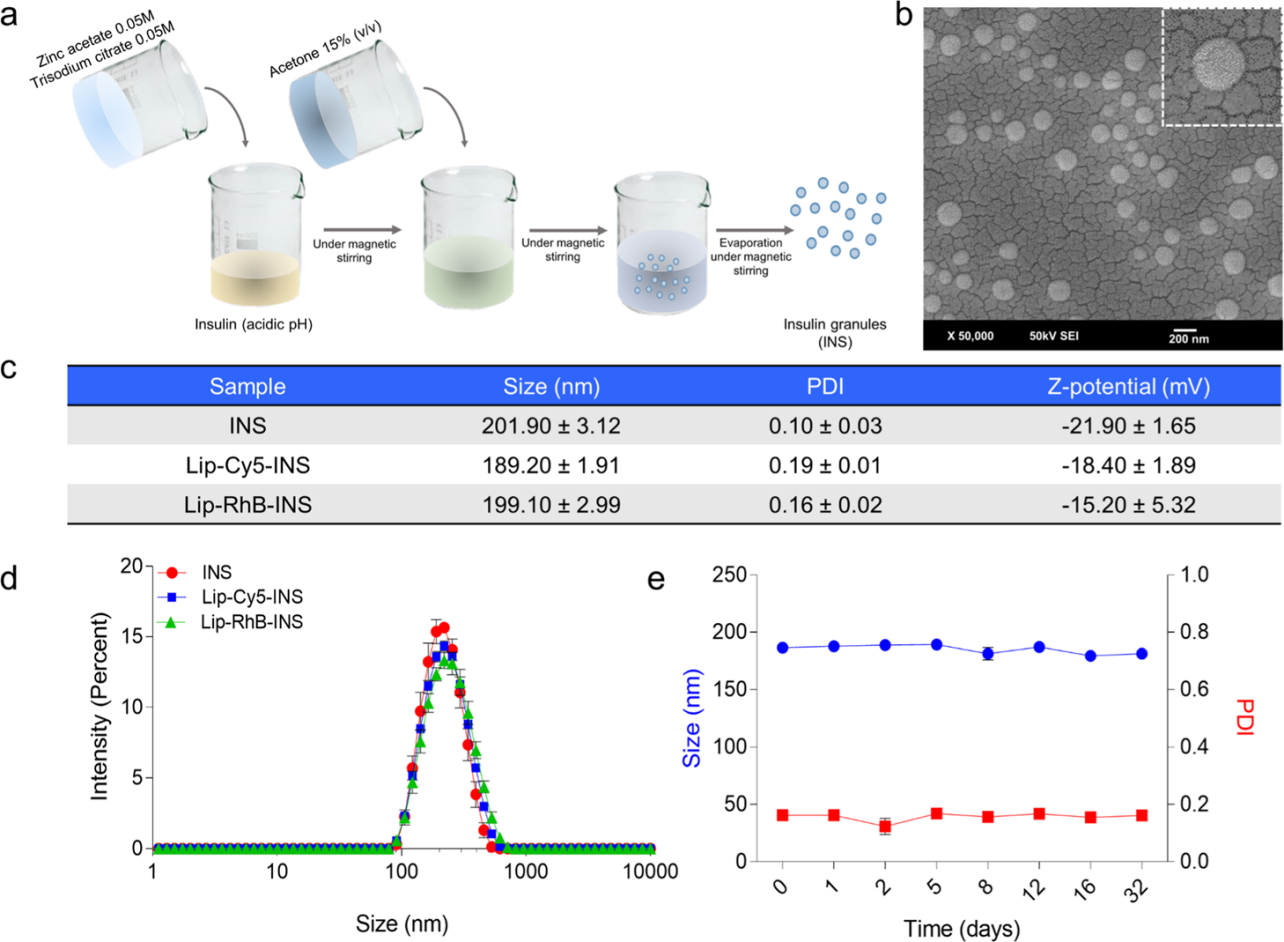
Synthesis, physicochemical characterization, and stability of insulin granules (INS). (a) Schematic of the INS crystallization process. (b) SEM image of INS. (c,d) Size distribution, PDI, and surface charge of INS and fluorescent INS (loaded with Lip-Cy5 and Lip-RhB, called Lip-Cy5-INS and Lip-RhB-INS, respectively). (e) INS stability in DI water at 25 °C.

**Figure 2:**
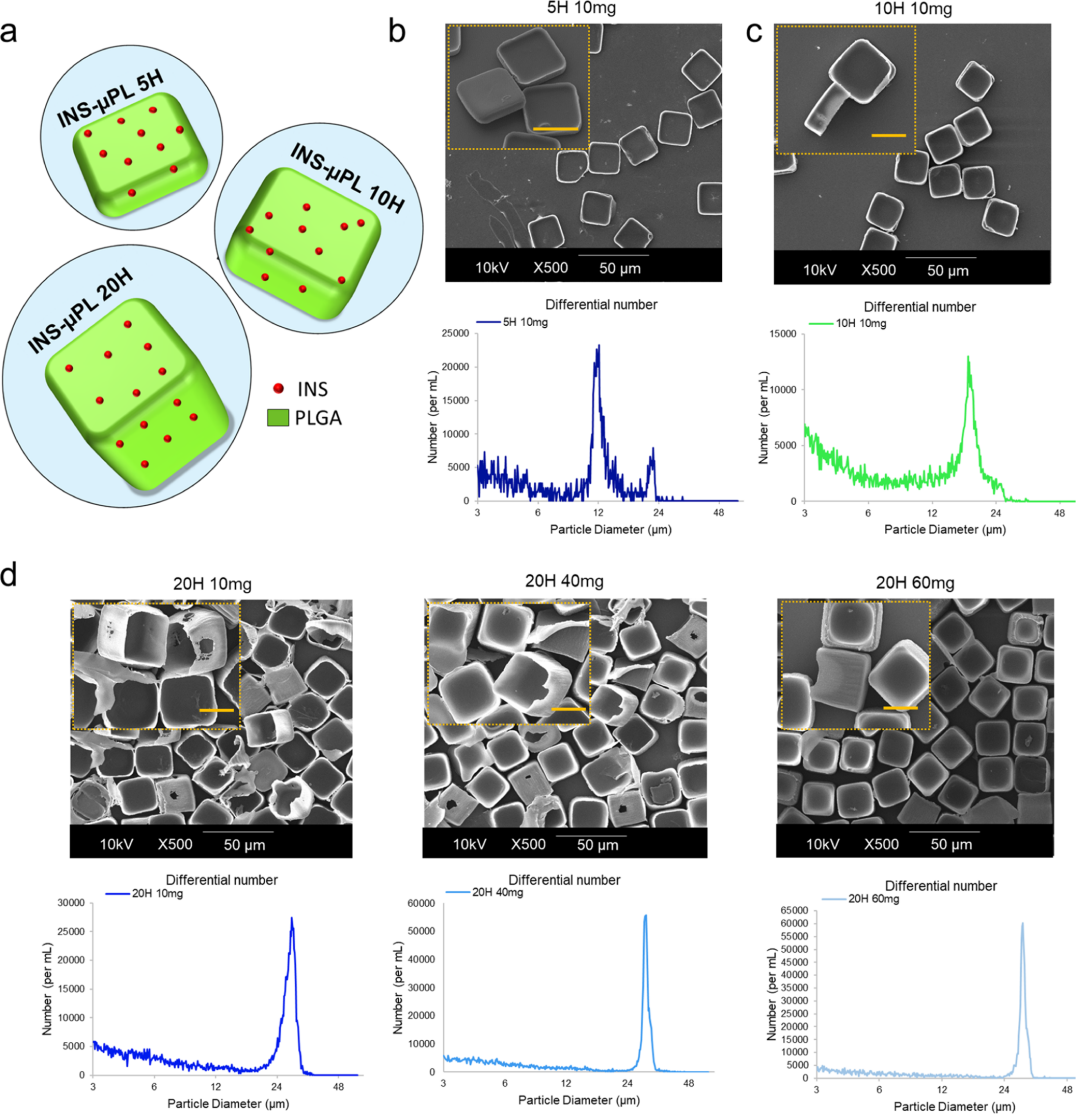
Geometrical characterization of different configurations of INS-μPLs. (a) Schematic representation of INS-μPLs with different thicknesses—5, 10, and 20 μm. SEM images and Multisizer Coulter Counter size distribution profiles for the (b) 5 μm thick INS-μPLs with 10 mg of PLGA (5H, 10 mg), (c) 10 μm thick INS-μPL with 10 mg of PLGA (10H, 10 mg), and (d) 20 μm thick INS-μPL made with 10, 40, and 60 mg of PLGA (20H, 10 mg; 20H, 40 mg; and 20H, 60 mg, respectively). The size bar in the insets of the SEM images is 20 μm.

The two main components in the INS-μPL are the insulin granules (INS) (Figure 1) and the prismatic PLGA matrix (Figure 2), containing and protecting the granules from rapid degradation (INS-μPL).

Inspired by the microcrystalline formulations which are typically adopted by the pharmaceutical industry,^32−34^ insulin granules were prepared using a crystallization process.^32^ Briefly, insulin (5 mg/mL) was dissolved in acidified water (HCl 10 mM, pH = 2.5), whereupon zinc acetate (0.05 M), trisodium citrate (0.05 M), and acetone 15% were added at room temperature under magnetic stirring (250 rpm) for 90 min. After the evaporation of the organic solvent, INS were collected by centrifugation (18,000*g* for 30 min) (Figure 1a). Under the scanning electron microscope, INS appeared as spherical nanoparticles with a characteristic size of about 200 nm (Figure 1b). These morphological features were confirmed by a dynamic light scattering (DLS) documenting an average size of 201.90 ± 3.12 nm and a monodisperse particle population with a polydispersity index (PDI) of 0.10 ± 0.03 (Figure 1c,d). INS granules showed a net negative surface charge of −21.90 ± 1.65 mV. In the absence of proteins, salts, and other biological molecules (DI water), INS were observed to be stable in size for at least 30 days at room temperature (Figure 1e). When a fluorescent probe, such as Lip-Cy5 and Lip-RhB, was dispersed in the insulin solution during crystallization and entrapped within the resulting nanoparticles, the fluorescent INS (Lip-Cy5-INS and Lip-RhB-INS) showed no significant difference in the average size (189.20 ± 1.91 and 199.10 ± 2.99 nm, respectively) and surface charge (−18.40 ± 1.89 and −15.20 ± 5.32 mV, respectively), as compared to the native insulin granules (Figure 1c,d).

In order to protect the granules from an excessively rapid dissolution and thus provide a sustained insulin release, INS were entrapped in microscopic, biodegradable PLGA porous matrices [microPlates (μPLs)] to realize the INS-loaded μPLs (i.e., INS-μPLs). INS-μPLs were obtained using a replica molding multi-step, top-down fabrication process.^35,36^ Briefly, a direct laser writing process was adopted to realize silicon master templates with an array of 20 × 20 μm squared wells whose depth can range from a few microns to several tens of microns to modulate the thickness of the resulting microparticles. These master templates were replicated into polydimethylsiloxane (PDMS) templates which were then replicated into a sacrificial PVA templates (Figure S1). The wells in the PVA template were carefully loaded with a mix of PLGA and INS (160 μg) (see Table S1 for details). The PLGA content in the paste can be modified to realize μPLs with different levels of compactness. Eventually, the resulting prismatic INS-μPLs were recovered via centrifugation after dissolution in water of the PVA templates. To accurately control and modulate the dissolution of the INS and the consequent release of insulin, five different configurations of INS-μPLs were realized with different thicknesses and PLGA concentrations. Specifically, these include 5 μm thick μPL with 10 mg of PLGA (5H INS-μPL), 10 μm thick μPL with 10 mg of PLGA (10H INS-μPL), and 20 μm thick μPL with either 10, 40, or 60 mg of PLGA (20H INS-μPL).

**Figure 3:**
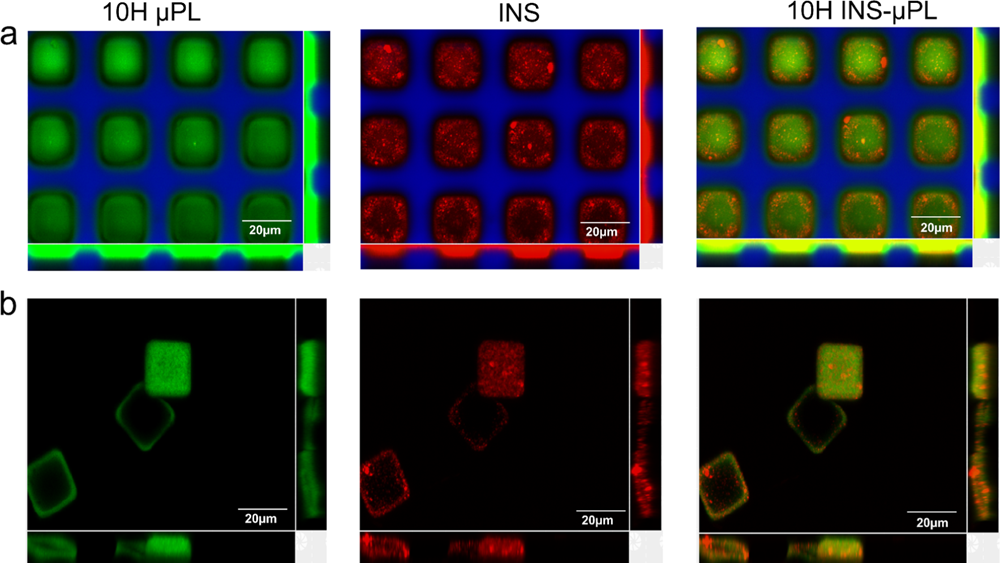
Representative images showing the distribution of insulin granules loaded in 10H INS-μPL. (a) Confocal images of RhB-loaded PVA templates (blue) containing μPL carrying free Curcumin (green) and Lip-Cy5-INS (red) and (b) confocal images of INS-μPL carrying free Curcumin (green) and Lip-Cy5-INS (red).

The morphology of the μPL is documented by scanning electron microscopy (SEM) images and Multisizer Coulter Counter analysis, as shown in Figure 2b–d. Note that, both the 5H and 10H INS-μPL made with 10 mg of PLGA appeared as completely and uniformly filled by the original polymer paste, whereas the 20H INS-μPL realized with lower amounts of PLGA (10 and 40 mg) presented holes in the structure. These imperfections result from an insufficient amount of PLGA. Differently, the 20H INS-μPL realized with 60 mg of PLGA appeared intact as the smaller 5H and 10H INS-μPL (Figure 2). Indeed, bigger particles require a larger amount of polymeric paste to be completely and uniformly filled. Multisizer analysis shows a peak between 10 and 15 μm for the 5H INS-μPL, around 20 μm for the 10H INS-μPL, and around 30 μm for the 20H INS-μPL. To note that, because of the squared shape of the μPL, the multisizer gives an average characteristic size rather than the actual height (5, 10, or 20 μm) or base (20 μm) lengths.^35,36^ Furthermore, representative confocal images (Figure 3) of the 10H INS-μPL PVA template and 10H INS-μPL demonstrate the uniform size and shape of the μPL before and after release from the PVA template and the quite uniform distribution of the insulin granules within the μPL polymeric matrix. Note that for generating these images, the INS were loaded with the infra-red fluorescent complex Lip-Cy5 (red dots—Figure 3), and the PVA template was loaded with the red fluorescent complex RhB (blue background—Figure 3). The top-down fabrication approach used to realize the INS-μPL does not require any chemical reaction between the polymer and INS and does not involve any polymerization process (i.e., cross-linking of polymers). As such, no additional organic solvents and elevated temperatures are used during the fabrication process, thus preserving more efficiently the stability of insulin.

### Encapsulation Efficiency, Release Rates, and Stability of INS-μPLs

**Figure 4:**
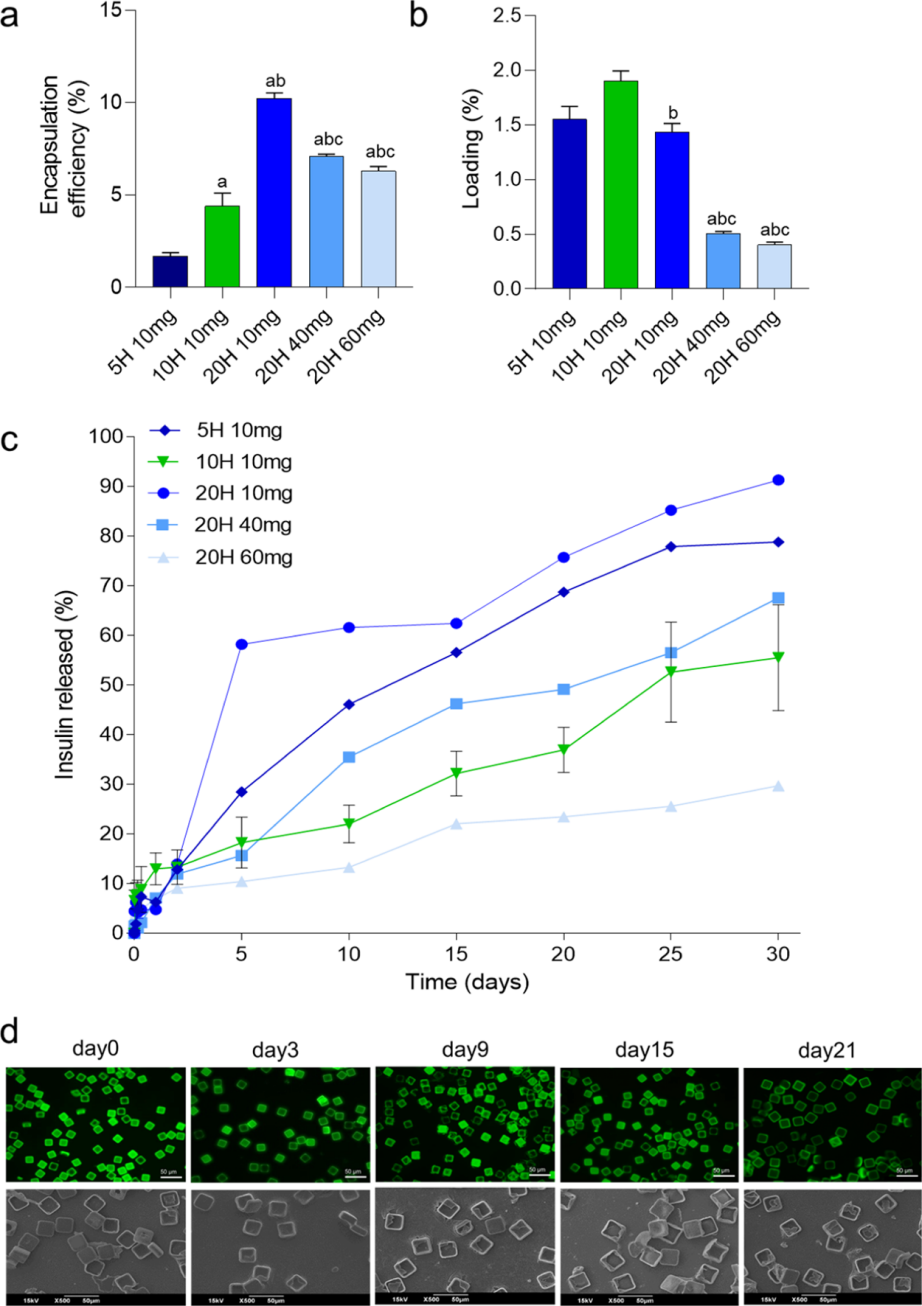
Biopharmaceutical characterization and in vitro insulin release profiles. (a,b) EE and loading and (c) in vitro insulin release profiles from INS-μPL made with different thicknesses of the μPL (5, 10, and 20 μm) and PLGA concentrations (10, 40, and 60 mg) under physiological conditions (PBS, pH = 7.4 at 37 °C) for 30 days (*n* = 3). (d) Fluorescence microscopy and SEM images of μPLs incubated under physiological conditions (PBS, pH = 7.4 at 37 °C) up to 21 days (*n* = 3), confirming the morphological stability of the PLGA microparticles. Results are expressed as average ± SEM. Statistical significance was determined by the one-way ANOVA post hoc Tukey test: ^a^*p* < 0.05 5H 10 mg vs 10H 10 mg, 20H 10 mg, 20H 40 mg, and 20H 60 mg; ^b^*p* < 0.05 10H 10 mg vs 20H 10 mg, 20H 40 mg, and 20H 60 mg; ^c^*p* < 0.05 20H 10 mg vs 20H 40 mg and 20H 60 mg.

To assess the insulin encapsulation efficiency (EE) and loading, INS-μPLs were loaded with 160 μg of the INS initial input per PVA template. As reported in Figure 4a, the encapsulation was higher using 20H INS-μPL, resulting into an EE = 10.23 ± 0.3, 7.11 ± 0.10, and 6.3 ± 0.2% of insulin for the 10, 40, and 60 mg of 20H INS-μPLs, respectively. The EE was 4.4 ± 0.7 and 1.7 ± 0.18% for the 10H and 5H INS-μPLs, respectively. However, the loading resulted to be higher for INS-μPL made with lower PLGA amounts (Figure 4b). The EE and loading could be largely improved for all tested configurations by performing a systematic optimization of all the different factors affecting the dispersion of the polymeric paste, enriched with the insulin granules, over the PVA template and the actual filling of the wells in the same template.^36^ These factors are the viscosity of the polymeric paste, the INS initial inputs, the spreading technique, the separation distance among the wells in the PVA template, the environmental conditions (humidity and temperature), and the PVA concentration in the sacrificial template. However, this systematic analysis transcends the objective of this work which provided an initial proof of the INS-μPL efficacy in managing T1DM.

More importantly, the insulin release profile was evaluated up to 1 month under physiologically relevant conditions [0.5 mL of phosphate-buffered saline (PBS) at pH = 7.4, 37 °C], mimicking the volume associated with an intra-tissue deposition of the μPL (Figure 4c). The mass of PLGA and the μPL height (geometry) played a major role in modulating the insulin release profile. μPL realized with larger PLGA amounts were generally associated with lower release rates. This is evident by comparing the 20H INS-μPL made with different amounts of PLGA. The 20H INS-μPL made with 10 mg and 40 mg of PLGA, which appeared to be largely damaged with empty spots and passing holes, resulted in faster release rates—91.3 ± 0.32 and 67.6 ± 0.58% insulin released at 30 days, respectively, as compared to intact 20H INS-μPLs made with 60 mg—29.7 ± 0.58% insulin released at 30 days (Figure 4c). The damaged 20H INS-μPLs were not further considered in the study. As per the effect of the μPL size, the direct comparison between the 5H and 10H INS-μPLs, both realized with the same 10 mg of the PLGA amount, is informative. The thinner microparticles presented an overall faster release rate with 78.8 ± 0.1% of the insulin being released from the 5H INS-μPLs at 30 days, as opposed to the 55.6 ± 18.5% measured for the 10H INS-μPLs (Figure 4c). In line with previous studies by the authors,^35−39^ it was here hypothesized that insulin could be released from the μPL upon dissolution of INS and the progressive diffusion of the molecular insulin out of the PLGA matrix. Based on this, we selected the 10H INS-μPL as the best configuration for the functional and in vivo studies as it provides an intermediate release profile: sufficiently faster than the 20H INS-μPL to possibly prevent hyperglycemic conditions within the first hours of the application; sufficiently slower than the 5H INS-μPL to guarantee a sustained insulin release for at least 30 days (Figure 4c). For the 10H INS-μPL configuration, the insulin release kinetics was also studied under hyperglycemic (400 mg/dL glucose) and normoglycemic (100 mg/dL glucose) conditions, documenting no statistically significant dependence of the release rates on the environmental conditions (Figure S2).

Moreover, in order to verify the progressive μPL matrix degradation, 10H INS-μPLs loaded with the natural green dye Curcumin were obtained and characterized over time (1 month) to assess any significant morphological change (Figure 4d). SEM and fluorescent microscopy images were obtained at predetermined time points after INS-μPL exposure to physiological conditions (0.5 mL of PBS, pH = 7.4 at 37 °C) and confirmed that the typical μPL prismatic shape with a 20 × 20 μm base was preserved for long incubation periods. This proves that, within the 1 month observation time, insulin release is largely affected by the progressive dissolution of insulin granules and diffusion of molecular insulin out of the μPL matrix into the surrounding aqueous environment.^36,37^ It is just important to note that differently from several other conventional PLGA microparticles, the μPLs exhibit a less compact structure that would allow water molecules to slowly permeate into the matrix without requiring extensive hydrolysis of the constituting polymer. Indeed, μPL stability analysis (Figure 4d) did show quite intact microparticles even after 21 days of exposure to a physiological solution. As such, the INS closer to the μPL surface would more rapidly feel the presence of the extra-particle physiological solution, degrade, and release insulin in the surrounding environment. Based on the above reasonings, 10H INS-μPL would act as a local drug depot and could be considered as artificial β cells.

### Biological and Cytotoxic Activities of 10H INS-μPL on Cells

**Figure 5:**
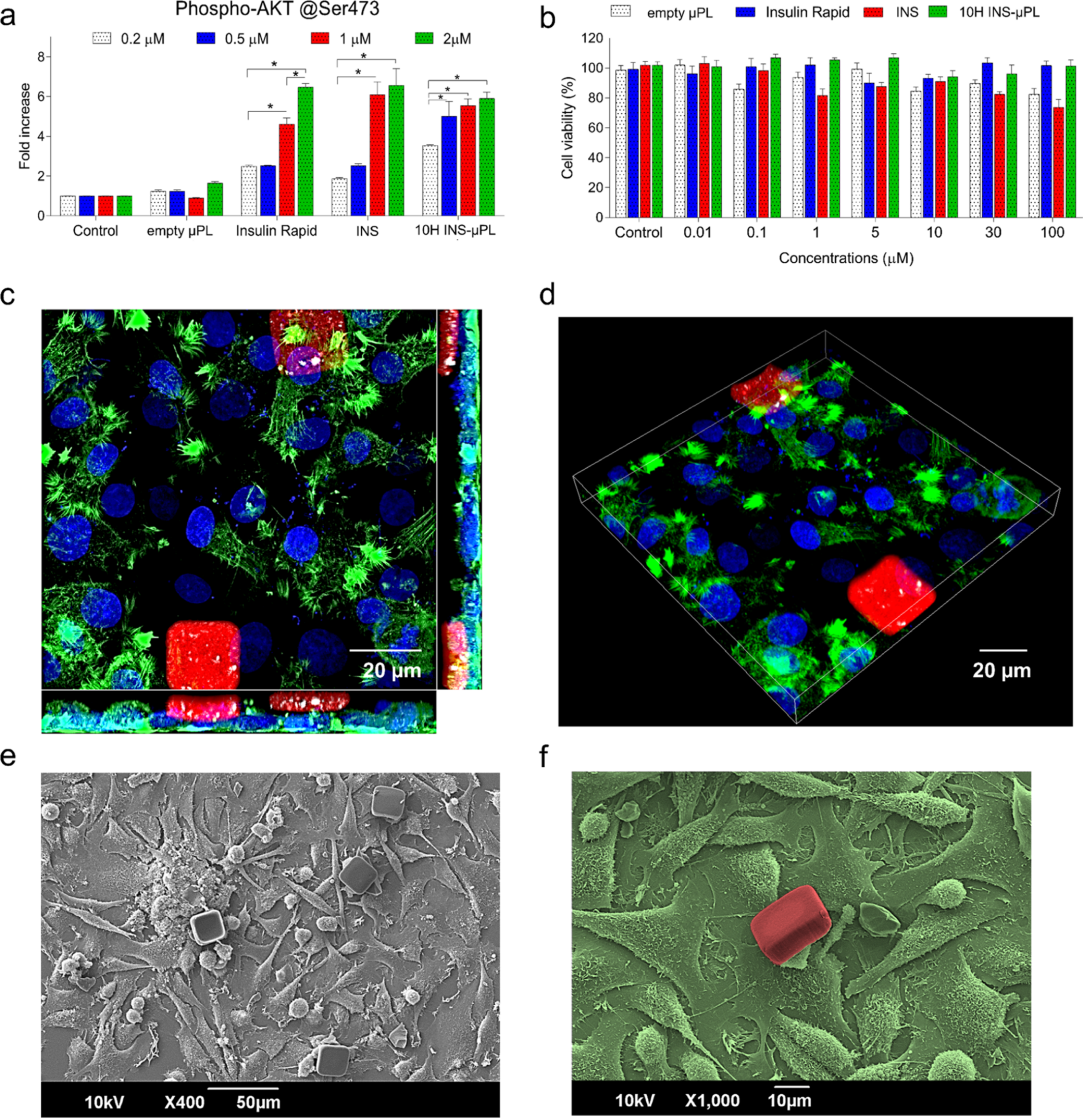
Biological activity and toxicity of 10H INS-μPL. (a) Biological activity of INS-μPLs, as compared to free INS, Insulin Rapid, and empty-μPL, on L6 cells proved through the analysis of AKT phosphorylation at Ser473. (b) Cytotoxicity of 10H INS-μPL, free INS, Insulin Rapid, and empty-μPL at different concentrations (0.01–100 μM) assessed on L6 cells. (c,d) 2D and 3D confocal microscopy images of L6 cells (blue = DAPI, green = phalloidin) at 24 h post-incubation with Lip-RhB labeled 10H INS-μPL. (e,f) SEM images of 10H INS-μPL at 24 h post-incubation with L6 cells. Results are expressed as the average ± SEM (*n* = 5). Statistical significance was determined by the one-way ANOVA post hoc Tukey test. * represents *p* < 0.05, and details of statistical analysis have been reported in Tables S2 and S3.

To demonstrate that insulin is still active even after entrapment into μPL, the amount of phosphorylated-AKT induced by the activation of insulin receptors was quantified (Figure 5a). Both 10H INS-μPL and INS were observed to increase the phosphorylation of AKT in a dose-dependent fashion (*p* < 0.05) (Figure 5a and Table S2). Notably, 10H INS-μPLs were more effective at low concentrations (0.5 μM), as compared to the molecular commercial insulin formulation (Insulin Rapid) returning, respectively, a 5.00 ± 1.3 and 2.52 ± 0.1-fold increase in AKT phosphorylation, as compared to the control (Figure 5a). At higher concentrations, all tested groups returned similar AKT phosphorylation. As expected, no effect was observed using empty-μPL (Figure 5a and Table S2). Furthermore, 10H INS-μPL showed no significant cytotoxic effect for the tested concentrations (0.01–100 μM) (Figure 5b, Table S3), while confocal and SEM images confirmed no visible effects on the cell morphology for up to 24 h (Figure 5c–f). Insulin is an anabolic hormone which is stored in pancreatic β cells in the form of granules consisting of insoluble crystalline hexameric insulin.^40^ The secretion of insulin from β cells is stimulated by elevated glucose levels, such as those occurring after a meal.^41,42^ Upon secretion and release from β cells, these natural granules of insulin dissolve rapidly in the bloodstream so that the molecular insulin facilitates the uptake of glucose into muscles and adipose tissues. Likewise, our synthetic INS dissolves easily when in contact with physiological solution, such as PBS at neutral pH or blood, making insulin rapidly available. However, unlike conventional insulin that is chemically and structurally unstable, INS and 10H INS-μPL exhibit a good stability profile (30 days) without any alteration in the function of the insulin, as was readily demonstrated by quantifying the amount of AKT phosphorylation induced upon exposure to INS (Figure 5) in L6 muscle cells. Notably, molecular stability is one of the main limitations in the effective encapsulation of insulin in biodegradable polymeric microparticles, obtained using conventional bottom-up fabrication processes (i.e., emulsion/solvent removal techniques).^43−45^

### Therapeutic Efficacy of 10H INS-μPLs in Diabetic Mice

**Figure 6:**
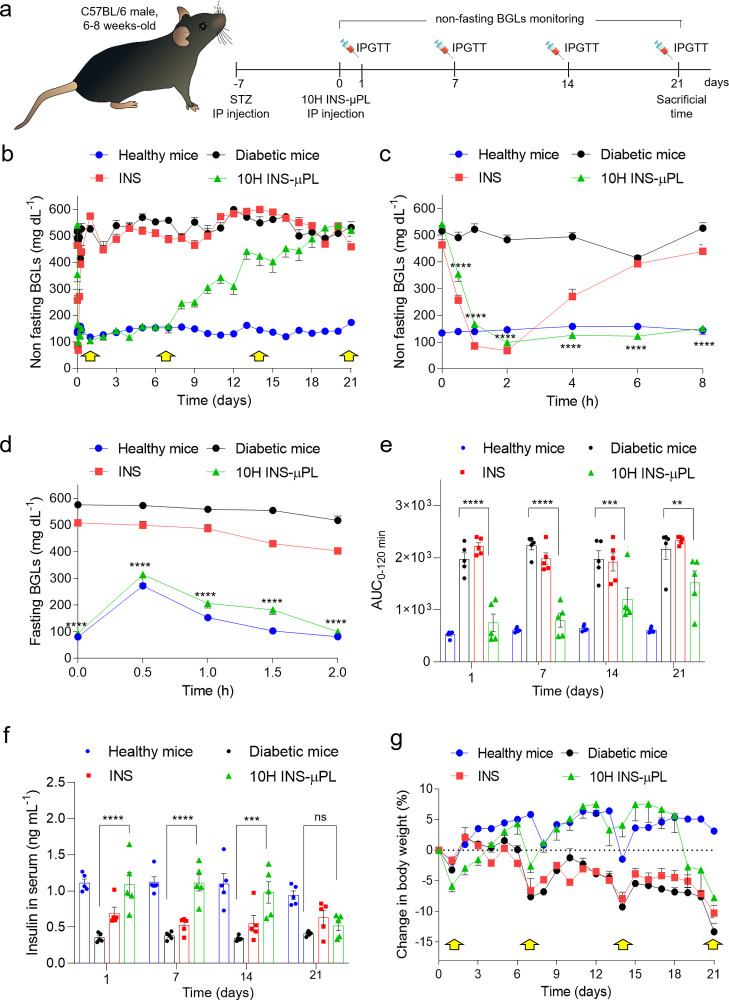
In vivo evaluation of 10H INS-μPL. (a) Experimental setup and timeline for the in vivo tests on C57BL/6 STZ-induced diabetic mice. (b) Non-fasting BGL measurements over 21 days post-ip deposition of 10H INS-μPL (yellow arrows indicate the IPGTT testing). (c) Non-fasting BGL measurements over the first 8 h post-ip deposition of 10H INS-μPL. (d) Fasting IPGTT at day 7 post-implantation of 10H INS-μPL. (e) Area under the BGL curve (AUC_0–120min_) from 0 to 120 min at different days (1, 7, 14, and 21) post-ip deposition of 10H INS-μPL. (f) Serum insulin levels at different days (1, 7, 14, and 21) post-ip deposition of 10H INS-μPL. (g) Change in body weight (%) (yellow arrows indicate the IPGTT). Results are expressed as the average ± SEM (*n* = 5). Statistical significance was determined by the two-way ANOVA post hoc Tuckey test. ** represents *p* < 0.01, *** represents *p* < 0.001, and **** represents *p* < 0.0001 for INS-μPL vs diabetic mice. Details of the statistical analysis are reported in Tables S4–S8.

STZ-induced type 1 diabetic C57BL/6 mice were chosen as a preclinical model for studying the capacity of the INS-μPL to normalize the BGLs in vivo. Diabetic mice were randomly assigned to different experimental groups and intraperitoneally injected with either INS or 10H INS-μPL with an insulin dosage of 0.05 and 0.5 IU/g body weight, respectively. Following the timeline of Figure 6a, BGLs of the treated mice were monitored by taking blood from the tail vein. Figure 6b,c provides the BGLs in mice treated with one administration only of 10H INS-μPL (green triangle); free INS (red squares); untreated diabetic mice (black circles); and healthy mice (blue circles). Within the first 2 h post-intraperitoneal (ip) deposition (Figure 6b), the BGLs in mice treated with 10H INS-μPL and INS were dramatically reduced compared to the untreated mice, reaching values comparable to that of healthy mice. Namely, the BGL values were 167.4 ± 49.0 mg/dL for the 10H INS-μPL-treated mice, 86.0 ± 12.4 mg/dL for the INS-treated mice, and 140.0 ± 9.2 mg/dL for the healthy mice, as opposed to 522.4 ± 46.3 mg/dL for the untreated mice. This would indicate a rapid release of insulin under elevated BGL conditions. Notably, the effect of INS in diminishing BGLs was stronger than that for the 10H INS-μPL due to the faster dissolution of the free INS, which are not protected by the μPL matrix, and the higher overall deposited insulin amounts (0.5 IU/g for INS vs 0.05 IU/g for 10H INS-μPL). Also, the restored normoglycemic conditions could not be supported longer than a few hours by the free INS, so that BGLs were observed to return to hyperglycemic conditions (393.4 ± 71.6 mg/dL) already at 6 h post-ip deposition. In contrast, only one administration of 10H INS-μPL was sufficient to provide glycemic control in vivo for almost 2 weeks. As shown in Figure 6c, the BGLs of mice treated with 10H INS-μPL started to rise only at day 7, returned at hyperglycemic conditions (>350 mg/dL) at day 13, and reached the untreated diabetic mice values at day 21.

An ip glucose tolerance test (IPGTT) was performed administering glucose at dosage 2 g/kg body weight in fasted mice at 1, 7, 14, and 21 days post-ip deposition of 10H INS-μPL to assess the BGL regulation capacity. At day 1 and 7, the treated diabetic mice efficaciously recovered the glycemic control after an initial increase in BGLs, and the BGLs were maintained normal (70–200 mg/dL) following the same temporal trend of the healthy mice (Figure 6d). Also, no statistically significant differences were observed between healthy and 10H INS-μPL-treated mice in terms of AUC_0–120min_ at day 1 (522.6 ± 27.6 vs 749.2 ± 167.4, respectively) and day 7 (609.8 ± 18.6 vs 799.9 ± 134.9, respectively) (Figure 6e). However, the same IPGTT conducted at day 14 and 21 post-implantation did not return equally satisfactory results both in terms of BGLs and AUC_0–120min_ (Figure 6e and Table S6). A significant increase in AUC_0–120min_ was observed when comparing healthy and INS-μPL-treated mice at day 14 (641.9 ± 26.2 vs 1203.3 ± 217.8, respectively—*p* < 0.05) and day 21 (606.7 ± 22.3 vs 1518.3 ± 225.9, respectively—*p* < 0.0001) post-implantation. Consistently with registered euglycemic levels, the serum insulin measurement by ELISA exhibited a release of insulin in mice treated with INS-μPL at day 1, 7, and 14, significantly higher compared to diabetic mice (1.10 ± 0.16 vs 0.35 ± 0.03 ng/mL; 1.11 ± 0.11 vs 0.38 ± 0.02 ng/mL; and 0.98 ± 0.15 vs 0.34 ± 0.02 ng/mL at day 1, 7, and 14, respectively—*p* < 0.0001), followed by a reduction in serum insulin at day 21 (0.52 ± 0.07 vs 0.41 ± 0.14 ng/mL) (Figure 6f and Table S7). Furthermore, mice treated with INS-μPL did not experience any significant change in the body weight, as compared to healthy mice for at least 18 days of treatment. In contrast, diabetic mice and mice treated with INS did undergo a significant weight loss starting already on day 5 (*p* < 0.05) (Figure 6g and Table S8).

Collectively, in vivo data show that mice treated with 10H INS-μPL maintain a full normoglycemic state for up to 7 days (<200 mg/dL) and return to a hyperglycemic state only at 13 days post-implantation (>350 mg/dL). Also, after ip infusion of glucose, 10H INS-μPLs are also able to successfully regain the glycemic control after an initial spike in BGLs at day 1 and 7 post-implantation, and this effect is slightly maintained at 14 days, and then, it disappears at 21 days post-implantation. The basal insulin release from 10H INS-μPLs is therefore not only sufficient to control the BGLs overtime in diabetic mice but somehow can also respond to glucose changes without provoking hypoglycemic crisis. Notably, this is occurring without using any glucose sensor but simply relying on the progressive degradation of the insulin granules entrapped within the PLGA matrix of μPL. It is important also to note that the effect of 10H INS-μPL in vivo appeared to be exerted over a shorter time scale, as compared to the in vitro release rates (only 30% of insulin released in 30 days) and particle degradation (Figure 4). This could be due to the combination of multiple factors including enzymatic reactions that could accelerate insulin release and PLGA degradation, as well as an effective larger release volume than that considered in vitro.^46,47^

## Conclusions

Hierarchically—structured polymeric microparticles carrying insulin granules were realized and pre-clinically validated in diabetic mice for the controlled and long-term release of insulin. 10H INS-μPLs are prismatic particles with a square base of 20 μm and a height of 10 μm capable of controlling the release of insulin for several weeks. A single ip administration of 10H INS-μPLs in STZ-diabetic mice reduced, already at 1 h post-implantation, the BGLs returned almost to the same value as that of healthy mice. Normoglycemic conditions were restored for about 2 weeks based on the solo-controlled degradation of the INS and the consequent sustained release of molecular insulin. Furthermore, mice implanted with 10H INS-μPL successfully regain glycemic control following the ip administration of a glucose bolus. In summary, these data show the potential of 10H INS-μPL as a promising platform for the treatment of T1DM with high and durable efficacy. Future studies will have to modulate the risk of hypoglycemia and extend further the temporal window of action of 10H INS-μPL through the addition of a highly specific biochemical sensor for BGL control.

## Experimental Section

### Materials

PDMS (Sylgard 184 and RTV615) was obtained from Dow Corning (USA). Poly(vinyl alcohol) (PVA), PLGA (50:50), MTT assay, trifluoroacetic acid, acetonitrile (ACN), STZ, zinc acetate, trisodium citrate, and paraformaldehyde were bought from Sigma-Aldrich (USA). Polycarbonate membrane filters were obtained from Sterlitech Corporation (USA). PBS, high-glucose Dulbecco’s modified Eagle’s minimal essential medium (DMEM), and fetal bovine serum (FBS) were obtained from GIBCO (Invitrogen Corporation, Italy). Curcumin (CURC) was purchased from Alfa Aesar (USA). l-α-Phosphatidylethanolamine-*N*-(lissamine rhodamine B sulfonyl) (ammonium salt) (Lip-RhB) was purchased from Avanti Polar, while Cy5-conjugated 1,2-distearoyl-*sn*-glycero-3-phosphorylethanolamine lipid chain (Lip-Cy5) was synthetized in our laboratory.

### Insulin Granules Synthesis, Physico–Chemical Characterization, and Stability

Insulin granules (INS) were prepared using a crystallization process, as reported in the literature with some modifications.^32^ Briefly, insulin (5 mg/mL) was dissolved in acidified water (HCl 10 mM, pH = 2.5), whereupon zinc acetate (0.05 M), trisodium citrate (0.05 M), and acetone 15% were added at room temperature under magnetic stirring (250 rpm) for 90 min. After the evaporation of the organic solvent, particles were centrifuged at 18,000*g* for 30 min, washed in water, and stored at 4 °C. The fluorescent insulin granules (INS) were obtained by adding 20 μL of a solution containing the fluorescent probe of interest, either Lipid-RhB (Lip-RhB) or Lipid-Cy5 (Lip-Cy5), to the mix comprising insulin (5 mg/mL) in acidified water, zinc acetate (0.05 M), trisodium citrate (0.05 M), and acetone 15% in the amber vial. After the evaporation of the organic solvent, the so-formed INS (Lip-RhB-INS or Lip-Cy5-INS, respectively) were collected by centrifugation at 18,000*g* for 30 min, washed in water, and stored at 4 °C.

Average size (nm), size distribution, and zeta potential (mV) of insulin crystals were analyzed using a DLS, as previously reported.^48^ However, the measurement of the ζ-potential was performed using a Smoluchowski constant *F* (ka) of 1.5 as a function of the electrophoretic mobility.

High-resolution scanning electron microscopy (JEOL JSM-7500 FA, Jeol, Tokyo, Japan) analysis was also performed to confirm the average size and evaluate the shape of the crystals. Briefly, a drop of insulin granules was put on a silica support, dried, and sputtered with gold/palladium for increasing the contrast and reducing the damage of the sample. SEM images were obtained with an acceleration voltage of 50 kV.

The insulin crystals yielding was quantified using ultra performance liquid chromatograph tandem mass spectrometry (Waters ACQUITY UPLC/MS). The mobile phase consisted of deionized water acidified with formic acid (FA) (0.1% v/v) (phase A) and ACN acidified with FA (0.1% v/v) (phase B), and the analysis was performed using an ACQUITY UPLC BEH C8 (50 × 2.1 mm, particle size 1.7 μm), and the following linear gradient was applied: 0–0.5 min: 20% phase B, 0.5–3.5 min: 20–100% phase B, 3.5–4.5 min: 100% phase B, 4.5–4.6 min: 100–20% phase B, 4.6–6.0 min: 20% phase B with a run time of 6 min and injection volumes of 2 μL. The mass spectrometer was run in the positive ESI mode, and insulin was measured by the single ion recording acquisition mode. The capillary and the cone voltages were set at 2.80 kV and 40 V, respectively. The source temperature was set at 125 °C, while the desolvation was set at 800 L/h with a temperature of 400 °C and a cone gas flow (N_2_) of 50 L/h, respectively. Data were obtained by MassLynx software and quantified by QuantLynx software.

The quantification of insulin was assessed using an external standard curve in a linear concentration ranging from 0.1 to 50 μg/mL. Insulin samples have been prepared in the matrix [ACN acidified with FA (0.1% v/v) and deionized water acidified with FA (0.1% v/v) at a ratio of 2:1 v/v], and the detection wavelength was fixed at 214 nm.

Furthermore, the stability of INS granules was assessed in water at room temperature for 32 days by DLS analysis of the samples at predetermined time points (0, 1, 2, 5, 8, 12, 16, and 32 days).

### Preparation of INS-μPLs

INS-loaded PLGA microPlates (INS-μPLs) were obtained via a multi-step replica molding process.^35,36^ First, three silicon master templates with a specific geometrical feature were made up via direct laser writing. These silicon master templates had wells squared in shape with an edge length of 20 μm, separated by 10 μm gap and a depth of 5, 10, and 20 μm for 5H INS-μPL, 10H INS-μPL, and 20H INS-μPL, respectively (Table S1). Then, the original master templates were replicated into PDMS templates using a mixture containing PDMS and a silicone elastomer curing agent (10:1, v/v). The replicas were then left under vacuum to remove bubbles, which were formed while mixing the PDMS with the curing agent, and polymerized at 60 °C for 4 h. The PDMS templates were peeled off the silica stub and used to obtain PVA templates by putting a PVA solution (10 w/v %) on their patterned surface. The resulting PVA films were dried at 60 °C to create the same arrays of wells as the original master templates. In the last step, the PVA templates were loaded with PLGA and INS (160 μg) dissolved in ACN, as reported in Table S1, to generate INS-μPLs. Then, the loaded PVA templates were dissolved in deionized water at room temperature in an ultrasonic bath, filtered in polycarbonate membrane filters (50 μm pore size), and the resultant INS-μPLs were collected via sequential centrifugation (2,000*g* for 5 min at 4 °C) and kept at 4 °C.

### Physico-Chemical Characterization of INS-μPLs

INS-μPLs were characterized using different methods. INS-μPL number, average size, and size distribution were analyzed using a Multisizer 4 Coulter particle counter (Beckman Coulter, CA). However, the square shape and size of INS-μPLs were confirmed after SEM analysis (Elios Nanolab 650, FEI). Specifically, a drop of the INS-μPL suspension was placed on a silica stub, dried, and homogeneously sputtered with gold to protect the sample from degradation and enhance the contrast. The images were obtained using an acceleration voltage of 5–15 kV.

Finally, confocal microscopy (Nikon A1, Milan) analysis was performed to assess the size, shape of INS-μPL, and also the distribution of INS through the μPL polymeric matrix. Briefly, Lip-Cy5 has been used to stain the INS (Lip-Cy5-INS), while Curcumin (CURC) has been used to stain the PLGA matrix of the particles.

### INS EE, Loading, and In Vitro Release Kinetics of INS-μPLs

To evaluate INS EE and loading, INS-μPLs were dissolved in ACN with FA (0.1% v/v) and deionized water with FA (0.1% v/v) in a ratio of 2:1 v/v. PLGA debris was removed after centrifugation (10,000*g* for 5 min at 4 °C), and the supernatant was analyzed by UPLC/MS, according to the chromatographic method reported in the above paragraph. The amount of insulin encapsulated in INS-μPL was calculated using a standard calibration curve obtained with samples having known insulin concentrations (0.1–50 μg/mL). EE and loadings were defined using the following equations
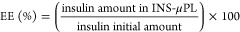
1
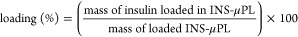
2An in vitro insulin release study was performed incubating INS-μPL in 500 μL of PBS solution (pH = 7.4) on an orbital shaker for 30 days, at 37 °C. At determined time points, samples were centrifuged (2,000*g* for 5 min at 4 °C), supernatants were removed, and pellets were destroyed with the mixture ACN acidified with FA (0.1% v/v) and deionized water acidified with FA (0.1% v/v) in a ratio of 2/1 v/v and analyzed via UPLC/MS.

### 10H INS-μPL Degradation Study

μPL matrix biodegradation was assessed by fluorescent microscopy (Leica 6000 microscope) and SEM analysis (SEM, Elios Nanolab 650, FEI). Empty μPLs (500 μL) were incubated in PBS (pH 7.4) under mechanical stirring at 37 °C for 1 month. Samples were analyzed to monitor changes in the shape and structure of the particles at predetermined time points (0, 3, 9, 15, and 21 days).

### In Vitro Biocompatibility and Biological Activity of 10H INS-μPL

The biocompatibility and the biological activity of INS-μPLs have been tested on skeletal muscle cells (L6 cells). In detail, L6 cells were cultured at 37 °C in 5% CO_2_, in high-glucose DMEM, supplemented with 15% FBS, 1% l-glutamine, and 1% penicillin/streptomycin. First, a cytotoxicity study was carried out on L6 cells at 24 h after incubation with INS and 10H INS-μPL at different concentrations (0.01, 0.1, 1, 5 10, 30, and 100 μM). Empty-μPL and a commercial rapid acting insulin form (i.e., Insulin Rapid) have been used as control groups. Specifically, empty-μPLs were used in amounts corresponding to the number of μPLs used for 10H INS-μPL, while Insulin Rapid was used at the same concentrations used for INS and 10H INS-μPL. L6 cells (1 × 10^4^ cells/well) were seeded into 96-well plates, and cells were treated with empty-μPL, Insulin Rapid, INS, and 10H INS-μPLs at the previous defined concentrations for 24 h. After 24 h of incubation, MTT assay was performed, as previously reported.^36^ The cell viability (%) was measured, as reported below
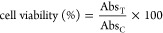
3where Abs_T_ is the absorbance of cells incubated with 10H INS-μPLs and Abs_C_ is the absorbance of cells used as the control group (non-treated cells).

The safety of 10H INS-μPLs was also confirmed through a microscope observation (SEM and confocal microscopy) of the interaction of L6 cells with 10H INS-μPL. Briefly, L6 cells were seeded on a glass slides pre-treated with fibronectin (1 mg/mL, Sigma-Aldrich). The day after, the cells were incubated with 10H INS-μPL (1 μM) for 24 h and treated, as previously reported.^39^ However, for confocal microscopy imaging, the samples were washed three times with PBS, fixed with 4% paraformaldehyde, and stained with 4',6-diamidin-2-fenilindolo (DAPI) and phalloidin (Figure 3) or wheat germ agglutinin (WGA) (Figure S3).

Furthermore, the biological activity of INS-μPL was assessed to prove the activation of the insulin receptor. Briefly, L6 cells (1 × 10^4^ cells/well) were seeded in 96-well plates and allowed to grow for 24 h. Cell serum starved overnight were treated with empty-μPL, Insulin Rapid, INS, and INS-μPL at different concentrations (0.2, 0.5, 1, and 2 μM) for 1 h. After treatment, cells were lysed, and phosphorylated AKT at Ser473 was assayed in accordance with the manufacturer’s protocol. The kit is designed specifically to quantify the activated (phosphorylated) AKT at Ser473 and/or total AKT.

### In Vivo Therapeutic Efficacy of 10H INS-μPL

In vivo studies were made in accordance with the regulations approved by our Institutional Animal Care and Use Committee at the Stanford University. We used male C57BL/6 mice at 6–8 weeks of age obtained from Charles River Laboratories, USA. To induce diabetes, each mouse was injected intraperitoneally with STZ (180 mg/kg). STZ was dissolved in disodium citrate buffer (pH = 4.5) at the concentration of 40 mg/mL. After STZ ip injection, the BGL of mice was measured daily for 7 days, and animals were considered diabetic when their non-fasting BGLs were >350 mg/dL. Briefly, the BGL was monitored daily by collecting blood (∼3 μL) from the tail vein of the mouse and measuring the BGLs using a Clarity GL2Plus glucose monitor.

Diabetic mice were intraperitoneally injected with a single administration of 10H INS-μPL or INS at an insulin dosage of 0.5 IU/g body weight and 0.05 IU/g body weight, respectively. In addition, two different groups of animals, no treated diabetic mice and healthy mice have been used as control groups. The non-fasting BGL was monitored at defined time points (0.5, 1, 2, 4, 6, and 8 h) right after the particle injection and daily in the following 21 days.

IPGTTs were assessed to evaluate the glucose responsiveness of INS-μPLs at predetermined time points (1, 7, 14, and 21 days). Mice were fasted overnight with free access to water before the ip administration of a bolus of glucose (2 g/kg). Briefly, BGLs were checked each 30 min for 2 h (0, 30, 60, 90, and 120 min) after injection, and the area under the curve (AUC_0–120min_) was also calculated.

Blood was collected from the tail vein of the fasted mice at the predetermined time points (1, 7, 14, and 21 days) for the measurement of serum insulin levels; serum was obtained by centrifugation of blood (3,000*g* for 5 min), and serum insulin levels were determined using a mouse insulin ELISA kit (Mercodia), according to the manufacturer’s protocol.

### Statistical Analysis

Data were expressed as means ± standard error of the mean, and all the in vitro and in vivo studies were performed in *n* = 5 (except when otherwise specified). The statistical significance was determined by one/two-way ANOVA post hoc Tukey test, and any difference was considered statistically significant when *p* < 0.05.

## Supporting Information Available

INS-μPL preparation; characterization of the microplate PVA template; in vitro insulin release profile from INS-μPL under physiological conditions at different glucose concentrations; confocal microscopy images of L6 cells at 24 h post-incubation with 10H INS-μPL; evaluation of total AKT; IPGTT at day 1, 14, and 21 post 10H INS-μPL ip deposition; stability of insulin in 10H INS-μPL overtime; and statistical analysis (PDF)
