# Supplementary material for: Insulin Granule-Loaded MicroPlates for Modulating Blood Glucose Levels in Type-1 Diabetes
Source: ACS Appl Mater Interfaces. 2021 Nov 9;13(45):53618–29. doi: 10.1021/acsami.1c16768 (PMC8603355; doi:10.1021/acsami.1c16768)
Supplement: Supplementary file 1 — am1c16768_si_001.pdf [file am1c16768_si_001.pdf]

Supporting Information

Insulin-granule loaded MicroPlates for Modulating Blood Glucose Levels in Type-1 Diabetes

Rosita Primavera[1,2], Elena Bellotti[1], Daniele Di Mascolo[1], Martina Di Francesco[1], Jing Wang[2], Bhavesh D. Kevadiya[2], Angelo De Pascale[3], Avnesh S Thakor[2#] and Paolo Decuzzi[1#] *

[1] Laboratory of Nanotechnology for Precision Medicine, Fondazione Istituto Italiano di Tecnologia, Via Morego 30, Genoa 16163, Italy

[2] Interventional Regenerative Medicine and Imaging Laboratory, Department of Radiology, Stanford University, Palo Alto, CA 94304, USA

[3] Unit of Endocrinology, Department of Internal Medicine & Medical Specialist (DIMI), University of Genoa, 16136 Genoa, Italy

[#] Avnesh Thakor and Paolo Decuzzi share the senior authorship

* Corresponding Author: Paolo Decuzzi, PhD – paolo.decuzzi@iit.it

Supporting Results

INS-μPL	PLGA (400 mg/mL)	INS	ACN
5H 10mg	25 μL	160 μg	37.5 μL
10H 10mg	25 μL	160 μg	37.5 μL
20H 10 mg	25 μL	160 μg	37.5 μL
20H 40 mg	100 μL	160 μg	50 μL
20H 60 mg	150 μL	160 μg	50 μL

Table S1. INS-µPL preparation.

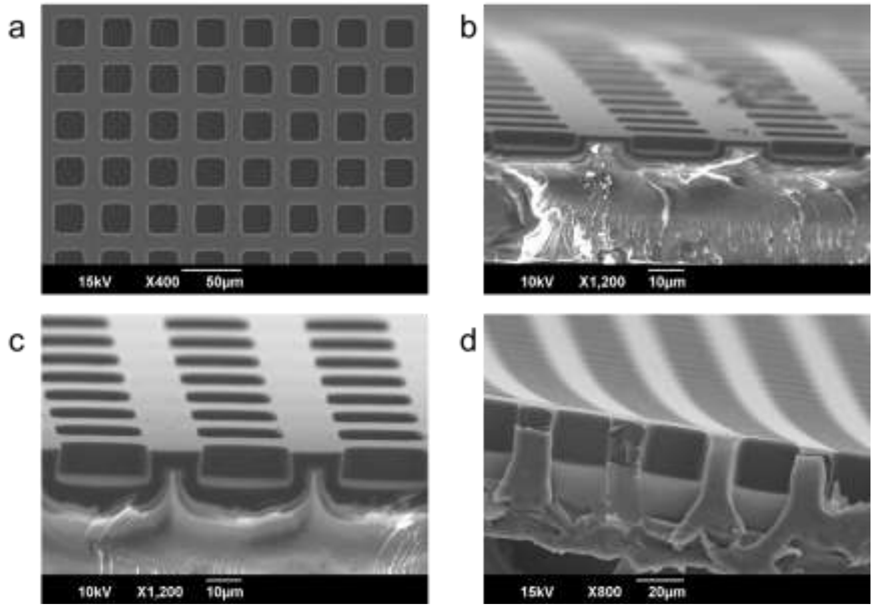

Figure S1. a. SEM images of the empty PVA template (20x20µm). **b-d.** Cross section of 5H **(a)**, 10H **(b)**, and 20H **(c)** templates.

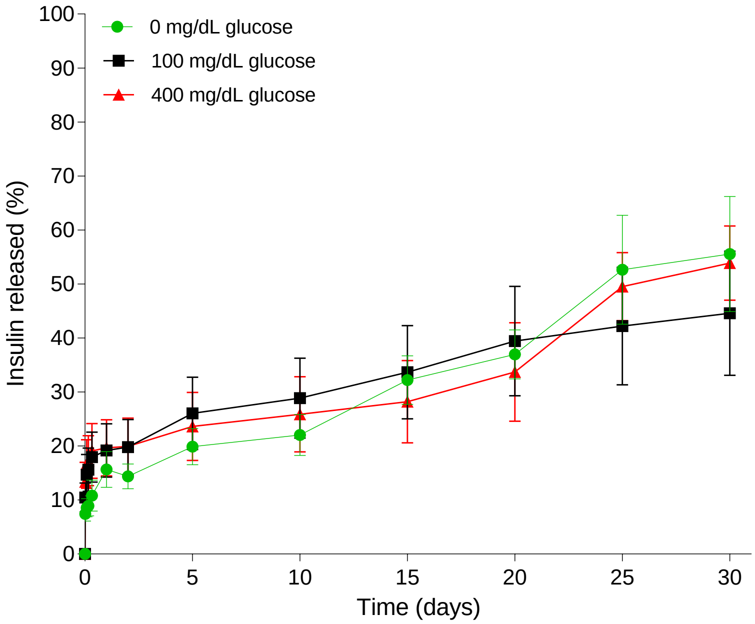

Figure S2. *In vitro* insulin release profile from 10H INS-µPL under physiological condition (PBS, pH = 7.4 at 37°C) at different glucose concentrations: 0, 100 mg/dL (normoglycemia condition) and 400 mg/dL of glucose (hyperglycemia condition).

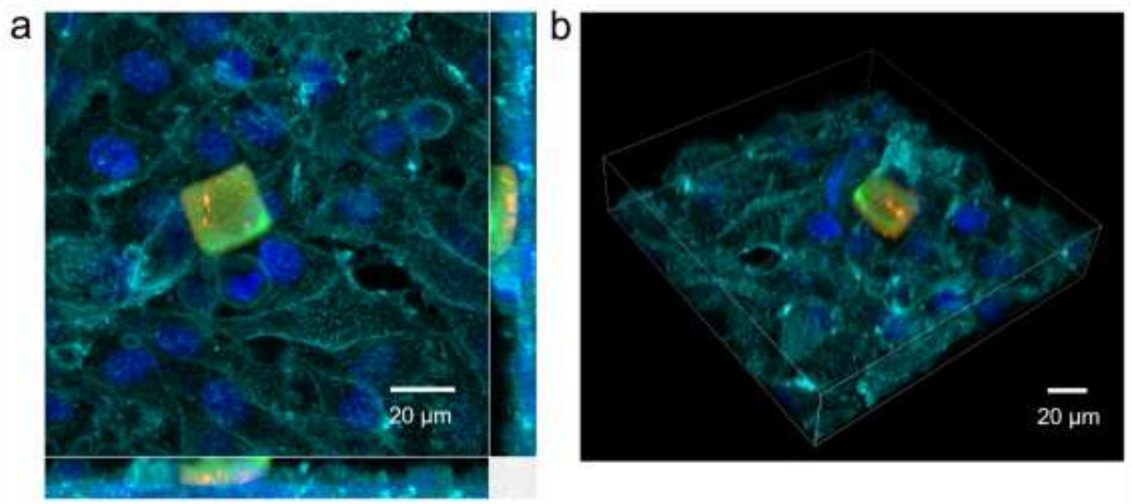

Figure S3. 2D and 3D confocal microscopy images of L6 cells (blu=DAPI, light blu=Wheat Germ Agglutinin, Alexa Fluor^{TM} 647 Conjugate) at 24h post incubation with Lip-Cy5 labeled INS (red) loaded into CURC-µPL (green).

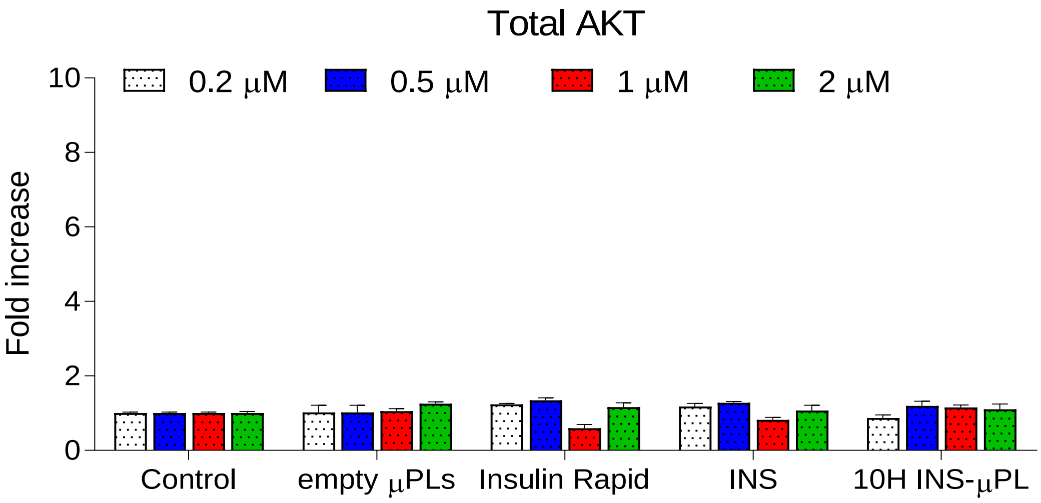

Figure S4. Evaluation of Total AKT at Ser473.

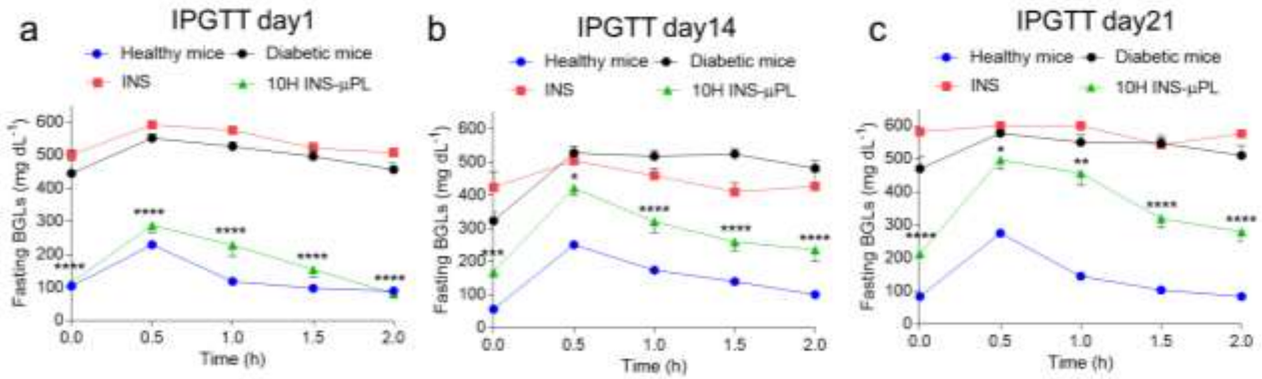

Figure S5. Intraperitoneal Glucose Tolerance Test (IPGTT) at **a**. day 1, **b**. day14, **c**. day21 post 10H INS-µPL intraperitoneal deposition. Results are expressed as the average ± SEM (n = 5). Statistical significance was determined by Two-way ANOVA post-hoc Tukey Test. **b**) * represents $p<0.05$, ** represents $p<0.01$, *** represents $p<0.001$ and **** represents $p<0.0001$ for 10H INS-µPL vs. diabetic mice. Details of the statistical analysis are in **Table S5**.

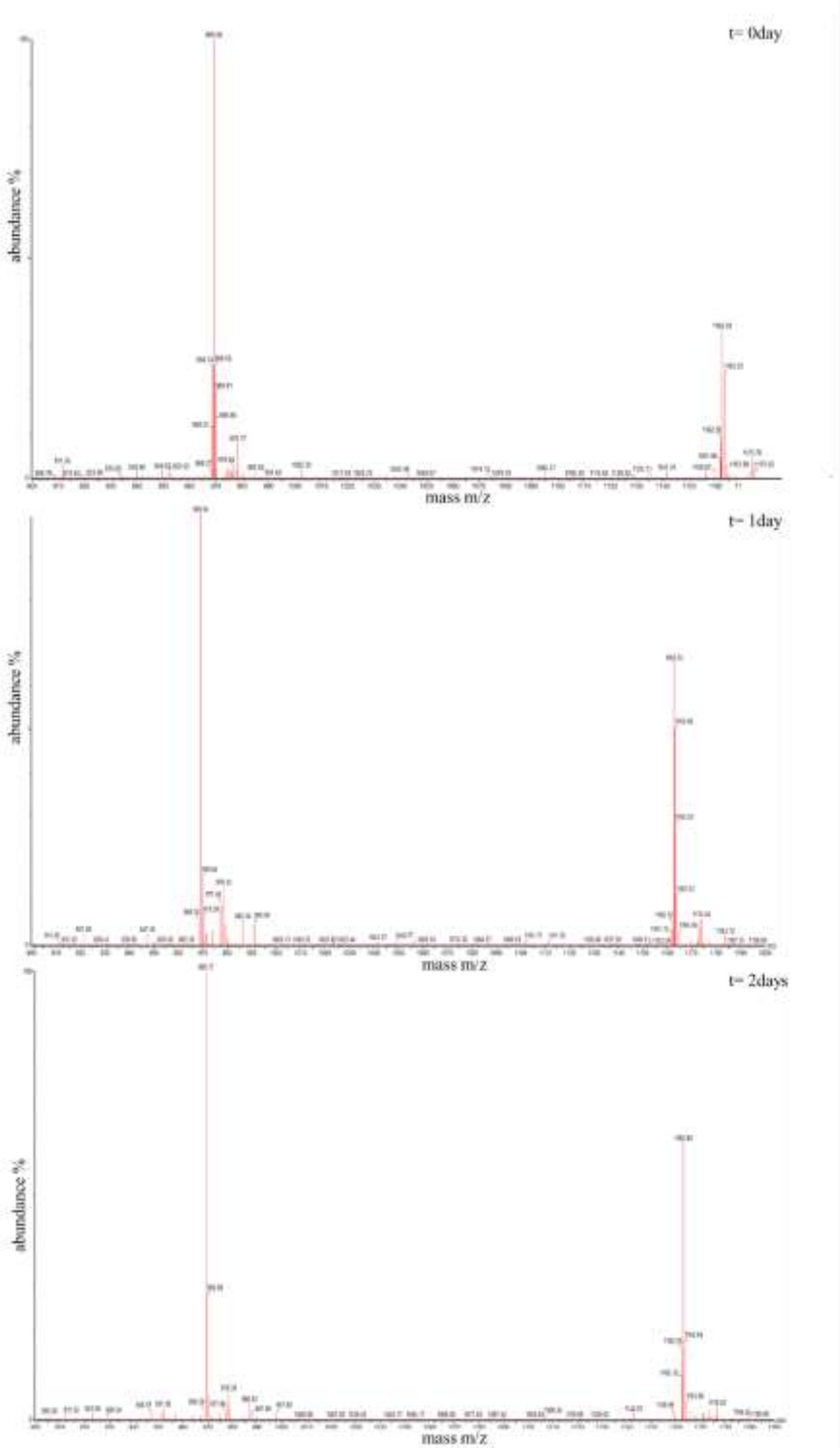

Figure S6. Mass spectrum graph showing the stability of insulin at 0, 1, and 2 days of incubation. Each bar is associated with an ion having a specific mass-to-charge ratio (m/z), while the length of the bar identifies the relative abundance (abundance %) of that ion.

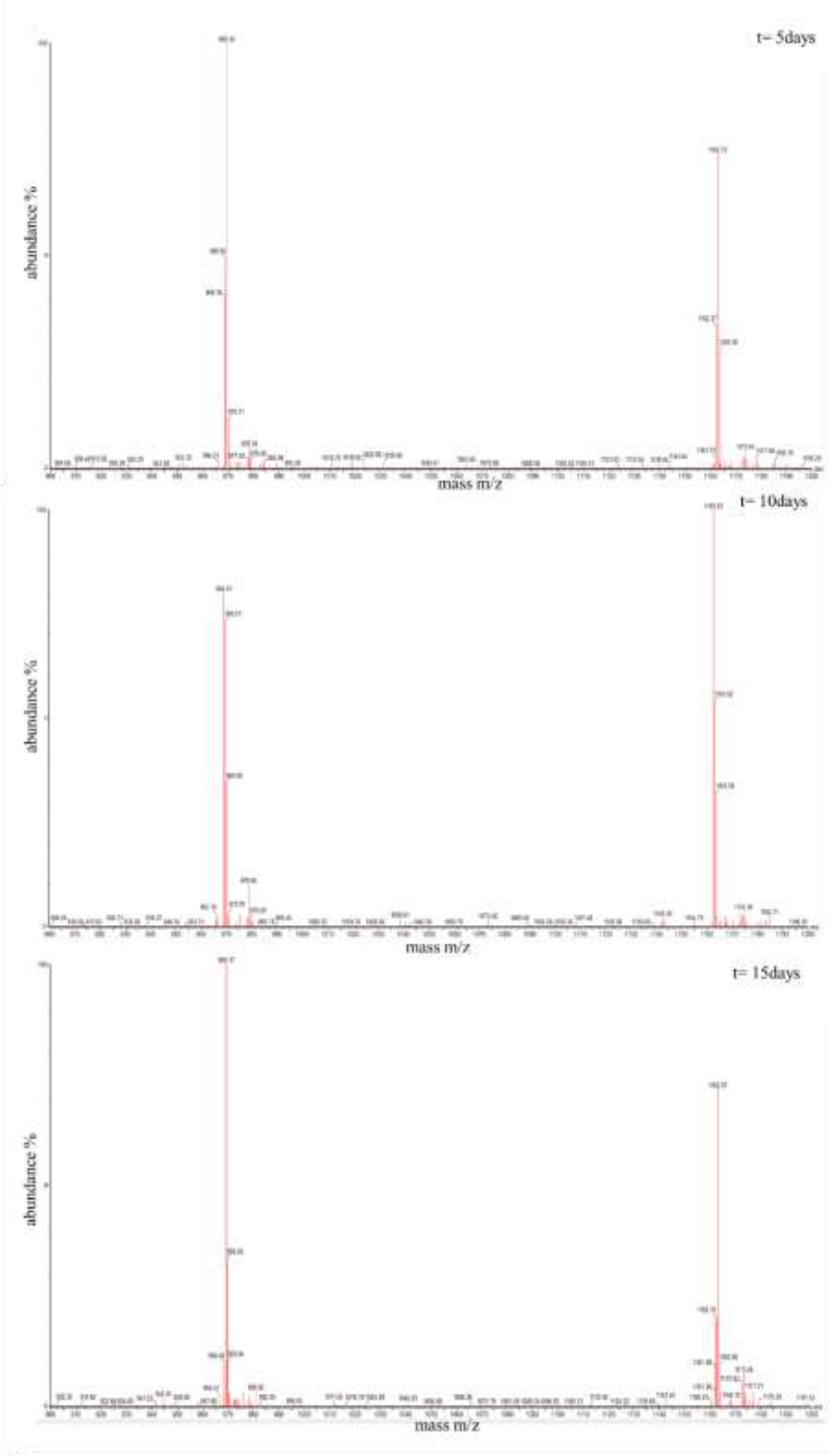

Figure S7. Mass spectrum graph showing the stability of insulin at 5, 10, and 15 days of incubation. Each bar is associated with an ion having a specific mass-to-charge ratio (m/z), while the length of the bar identifies the relative abundance (abundance %) of that ion.

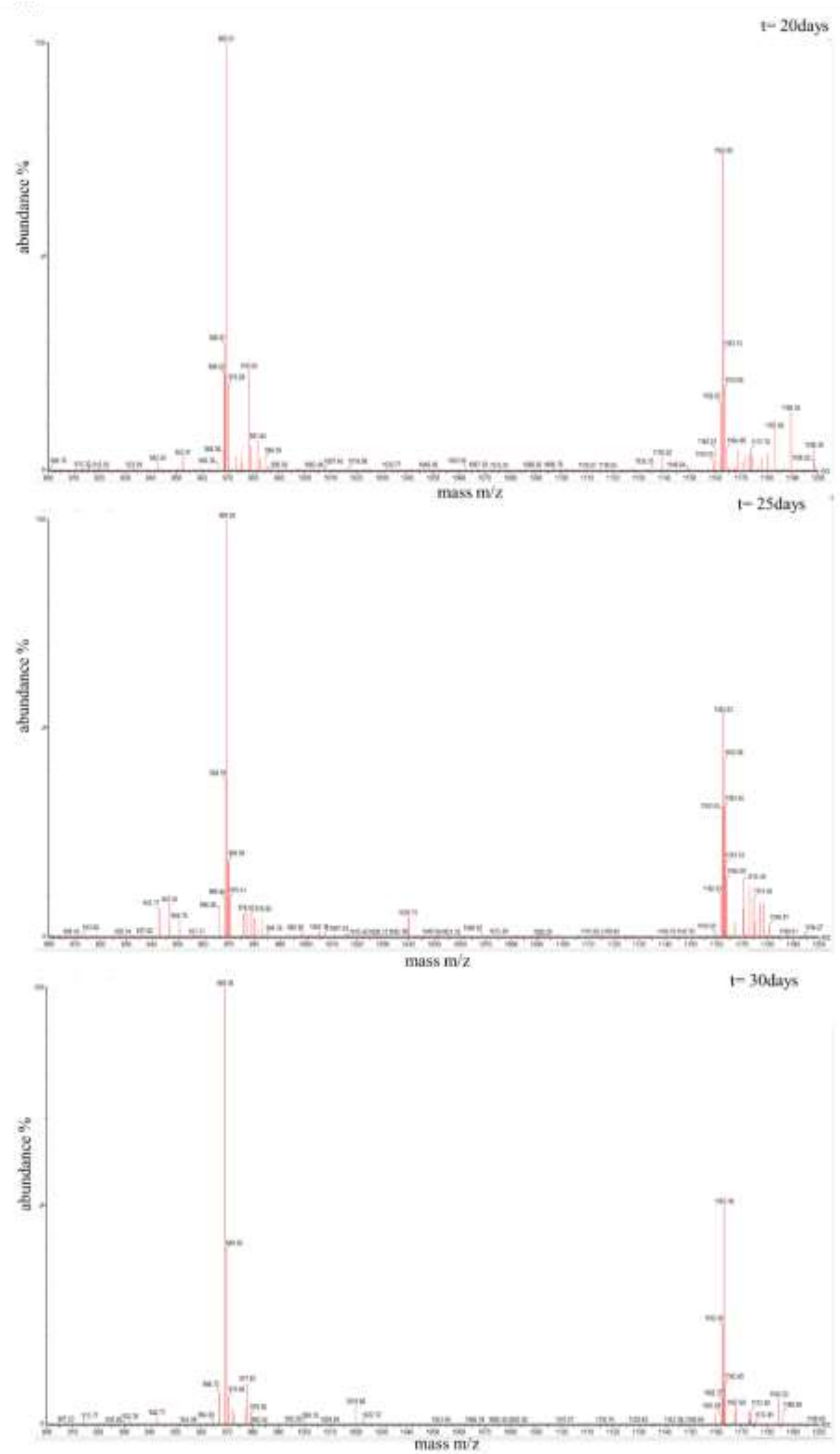

Figure S8. Mass spectrum graph showing the stability of insulin at 20, 25, and 30 days of incubation. Each bar is associated with an ion having a specific mass-to-charge ratio (m/z), while the length of the bar identifies the relative abundance (abundance %) of that ion.

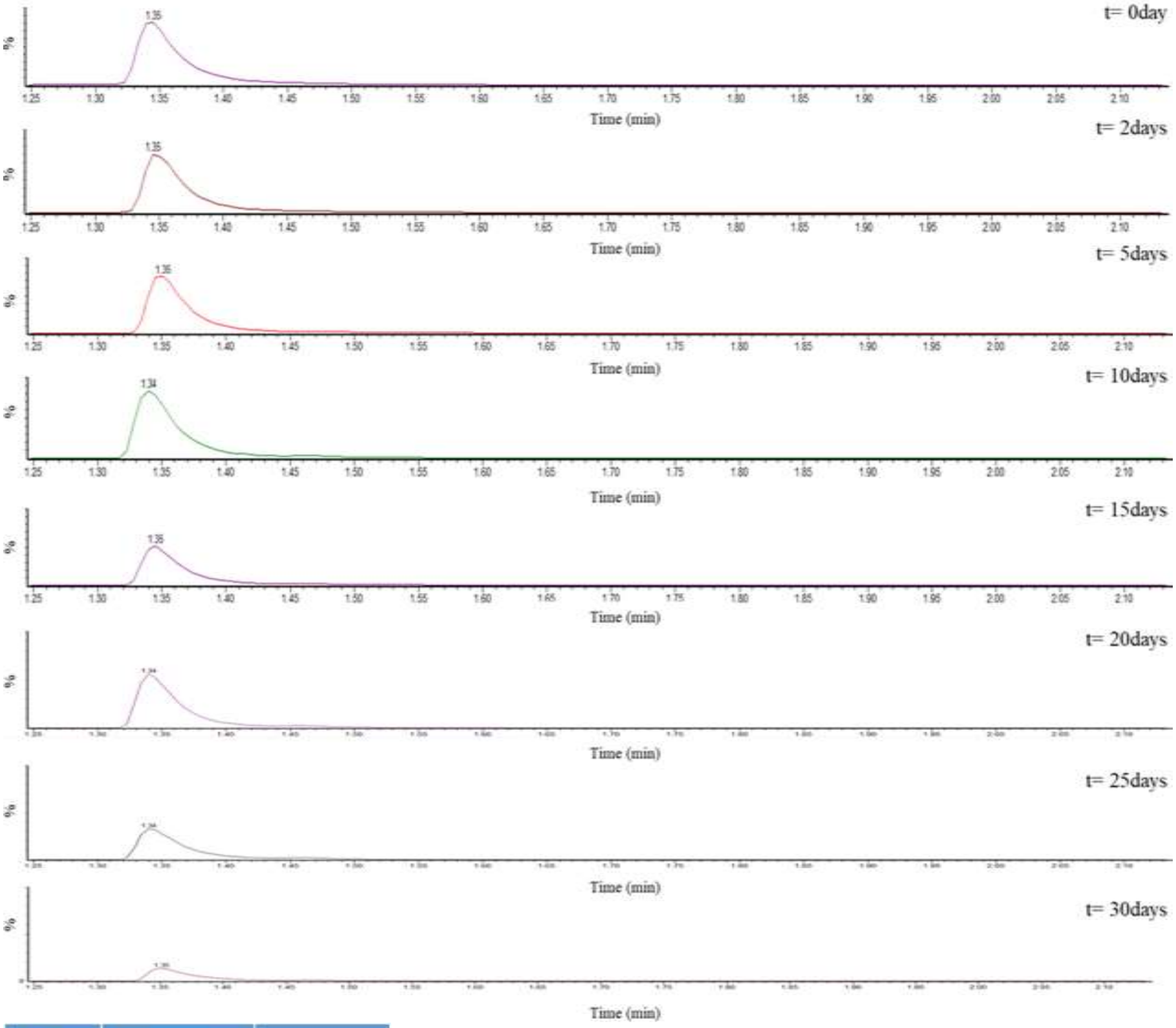

Time (days)	Retention Time (min)	Area
0	1.364	4300898.67
2	1.358	3850419.33
5	1.364	3711530
10	1.329	3636953.67
15	1.381	2396736
20	1.358	2361148
25	1.375	1854683.5
30	1.386	1090762.67

Figure S9. Chromatograms showing the stability overtime of insulin in the 10H INS-µPL. The graph shows the peak for insulin at predetermined time points. The table summarizes the retention time and Area values as a function of time.

Supporting Statistical Analysis

The statistical analysis (One/Two-way ANOVA post-hoc Tukey Test) is expressed as: p values < 0.05 (*), < 0.01 (**), < 0.001 (***) and < 0.0001 (****). "ns" is not statistically significant.

Experimental groups	0.2µM vs. 0.5µM	0.2µM vs. 1µM	0.2µM vs. 2µM	0.5µM vs. 1µM	0.5µM vs. 2µM	1µM vs. 2µM
Control	ns	ns	ns	ns	ns	ns
empty-µPL	ns	ns	ns	ns	ns	ns
Insulin Rapid	ns	***	****	***	****	***
INS	ns	****	****	****	****	ns
10H INS-µPL	*	***	****	ns	ns	ns

Table S2. Biological activity of 10H INS-µPL.

Insulin concentrations (µM)	empty-µPL vs. Insulin Rapid	empty-µPL vs. INS	empty-µPL vs. 10H INS-µPL	Insulin Rapid vs. INS	Insulin Rapid vs.10H INS-µPL	INS vs. 10H INS-µPL
0	ns	ns	ns	ns	ns	ns
0.01	ns	ns	ns	ns	ns	ns
0.1	*	ns	***	ns	ns	ns
1	ns	ns	ns	**	ns	***
5	ns	ns	ns	ns	*	**
10	ns	ns	ns	ns	ns	ns
30	ns	ns	ns	****	ns	ns
100	**	ns	**	****	ns	****

Table S3. Cytotoxicity of 10H INS-µPL, free INS, Insulin Rapid, and empty µPL at different concentrations (0.01-100 µM) assessed on L6 cells.

Time (day)	Healthy mice vs. Diabetic mice	Healthy mice vs. INS	Healthy mice vs. 10H INS-μPL	Diabetic mice vs. INS	Diabetic mice vs. 10H INS-μPL	INS vs. 10H INS-μPL
0	****	****	****	ns	ns	*
0.021	****	****	****	****	****	***
0.041	****	ns	ns	****	****	**
0.083	****	*	ns	****	****	ns
0.166	****	****	ns	****	****	****
0.25	****	****	ns	ns	****	****
0.33	****	****	ns	**	****	****
1	****	****	ns	ns	****	****
2	****	****	ns	ns	****	****
3	****	****	ns	ns	****	****
4	****	****	ns	ns	****	****
5	****	****	ns	ns	****	****
6	****	****	ns	ns	****	****
7	****	****	ns	*	****	****
8	****	****	**	***	****	****
9	****	****	***	**	****	****
10	****	****	****	ns	****	****
11	****	****	****	ns	****	****
12	****	****	****	ns	****	****
13	****	****	****	ns	****	****
14	****	****	****	ns	****	****
15	****	****	****	ns	****	****
16	****	****	****	ns	****	****
17	****	****	****	ns	ns	***
18	****	****	****	ns	ns	ns
19	****	****	****	ns	ns	ns
20	****	****	****	ns	ns	ns
21	****	****	****	ns	ns	ns

Table S4. Non-fasting blood glucose levels monitoring over 21 days after injection of 10H INS-μPL.

	Time (min)	Healthy mice vs. Diabetic mice	Healthy mice vs. INS	Healthy mice vs. 10H INS-µPL	Diabetic mice vs. INS	Diabetic mice vs. 10H INS-µPL	INS vs. 10H INS-µPLs
day1	0	****	****	ns	ns	****	****
	30	****	****	ns	ns	****	****
	60	****	****	****	ns	****	****
	90	****	****	ns	ns	****	****
	120	****	****	ns	ns	****	****
day7	0	****	****	ns	**	****	****
	30	****	****	ns	***	****	****
	60	****	****	*	***	****	****
	90	****	****	***	****	****	****
	120	****	****	ns	****	****	****
day14	0	****	****	**	*	***	****
	30	****	****	****	ns	*	ns
	60	****	****	***	ns	****	***
	90	****	****	**	**	****	***
	120	****	****	***	ns	****	****
day21	0	****	****	****	****	****	****
	30	****	****	****	ns	*	**
	60	****	****	****	ns	**	****
	90	****	****	****	ns	****	****
	120	****	****	****	ns	****	****

Table S5. Intraperitoneal Glucose Tolerance Test (IPGTT) at different times (day1, day7, day14, and day21).

$AUC_{0\text{-}120min}$	Healthy mice vs. Diabetic mice	Healthy mice vs. INS	Healthy mice vs. 10H INS-µPL	Diabetic mice vs. INS	Diabetic mice vs. 10H INS-µPL	INS vs. 10H INS-µPL
day1	****	****	ns	ns	****	****
day7	****	****	ns	ns	****	****
day14	****	****	*	ns	***	**
day21	****	****	****	ns	**	***

Table S6. Area under the curve (AUC) from 0 – 120min at different days (1, 7, 14, 21) post ip deposition of 10H INS-µPL.

Insulin in serum	Healthy mice vs. Diabetic mice	Healthy mice vs. INS	Healthy mice vs. 10H INS-µPL	Diabetic mice vs. INS	Diabetic mice vs. 10H INS-µPL	INS vs. 10H INS-µPL
day1	****	**	ns	*	****	*
day7	****	****	ns	ns	****	***
day14	****	***	ns	ns	***	ns
day21	***	ns	**	ns	ns	ns

Table S7. Insulin quantification in serum at different days (1, 7, 14, 21) post ip deposition of 10H INS-µPL.

Time (day)	Healthy mice vs. Diabetic mice	Healthy mice vs. INS	Healthy mice vs. 10H INS-µPL	Diabetic mice vs. INS	Diabetic mice vs. 10H INS-µPL	INS vs. 10H INS-µPL
0	ns	ns	ns	ns	ns	ns
1	ns	ns	ns	ns	ns	ns
2	ns	ns	ns	ns	*	*
3	ns	ns	*	ns	ns	ns
4	ns	*	ns	ns	ns	ns
5	ns	ns	ns	ns	ns	ns
6	*	***	ns	ns	ns	**
7	****	****	****	ns	*	ns
8	***	*	ns	ns	****	**
9	***	**	ns	ns	**	**
10	**	****	ns	ns	**	****
11	****	****	ns	ns	****	****
12	****	****	ns	ns	****	****
13	****	****	ns	ns	***	****
14	***	**	*	ns	****	****
15	****	***	ns	ns	****	****
16	****	****	ns	ns	****	****
17	****	****	ns	ns	****	****
18	****	****	ns	ns	****	****
19	****	****	***	ns	ns	ns
20	****	****	****	ns	ns	ns
21	****	****	****	ns	*	ns

Table S8. Change in body weight (%) over 21 days post ip deposition of 10H INS-µPL.
